# Measures of clinical effect in vestibular rehabilitation

**DOI:** 10.1055/s-0045-1806831

**Published:** 2025-10-31

**Authors:** Gabriela Ramos Ventura, Maristela Linhares dos Santos, Joubert Vitor de Souto Barbosa, Emannuel Alcides Bezerra Rocha, Gerônimo Bouza Sanchis, Rafael Limeira Cavalcanti, Marcello Barbosa Ottoni Gonçalves Guedes, Johnnatas Mikael Lopes

**Affiliations:** 1 Universidade Federal do Rio Grande do Norte, Centro de Ciências da Saúde, Departamento de Fisioterapia, Natal RN, Brazil.; 2 Universidade Federal do Rio Grande do Norte, Natal RN, Brazil.; 3 Universidade Federal de Minas Gerais, Belo Horizonte MG, Brazil.; 4 Marinha do Brasil, Natal RN, Brazil.; 5 Universidade Federal do Vale do São Francisco, Paulo Afonso BA, Brazil.

Dear Editor,

Peripheral vestibular hypofunction is a condition caused by the involvement of specific regions of the inner ear or the vestibular nerve, characterized by the functional loss of one or both peripheral vestibular systems, which can cause symptoms such as dizziness, vertigo, postural instability, oscillopsia, nausea, loss of balance, and proprioceptive deficit. 1 These symptoms can interfere with the quality of life of patients, making it difficult to perform daily activities. This dysfunction can increase the risk of falls, due to the impairment of balance and body perception, reinforcing the need for targeted strategies that promote functional recovery and prevent secondary complications. 1

Proprioceptive training has emerged as an alternative for the treatment of individuals with vestibular hypofunction, since it consists of using mechanical and somatosensory stimuli to improve skills that depend on sensorimotor integration, through exercises that challenge postural control and body perception. 2 This process is influenced by neural factors that are important for the consolidation of motor learning, such as the function of the cerebellum and primary cortex, which, when combined with different somatosensory stimuli, produce proprioceptive adaptations. 3

In this context, the study by Özaltın et al. 4 presented relevant findings regarding the vestibular rehabilitation of individuals with peripheral vestibular hypofunction. However, some issues related to the clinical usefullness of the results and their applicability, in addition to statistical significance, require further exploration to strengthen their practical implications.

When analyzing the data and calculating the measures of clinical effect, 5 it was observed that groups 1 and 2 present statistical similarity in some variables but generate distinct clinical effects. This may be attributed to a sampling problem, resulting in type-II error.

Measures of clinical effect were calculated, including Cohen's d, Cohen's U3, number needed to treat (NNT), and probability of superiority (PS). 5 In dynamic balance, assessed by the Tinneti Gait and Timed Up and Go (TUG) tests, Cohen's d values were 0.93 and −0.99, respectively, indicating a large clinical effect of group 1, compared to group 2. These results show that 82.4% (Cohen's U3) of the individuals in group 1 surpassed the mean of group 2 in the Tinneti Gait test, with a probability of superiority of 74.5% and an NNT of 2.98. Similar findings were observed for the TUG test ( Table 1 ).

**Table 1:** Measures of clinical effect in measurements related to vestibular function

		x _1_	x _2_	sd _1_	sd _2_	x _1_ - x _2_	SD _m_	Cohen's d	NNT	PS	Effect
Tinetti balance	Group 1 versus 2	15.1	11.2	0.87	2.69	3.9	1.78	2.19	1.41	93.9	Superior
	Group 1 versus 3	15.1	8.1	0.87	2.42	7	1.645	4.26	1.25	99.9	Superior
	Group 2 versus 3	11.2	8.1	2.69	2.42	3.1	2.555	1.21	2.18	81.2	Superior
Tinetti Gait test	Group 1 versus 2	11.6	10.8	0.69	1.03	0.80	0.86	0.93	2.98	74.5	Superior
	Group 1 versus 3	11.6	11	0.69	1.05	0.6	0.87	0.69	4.17	68.7	Non-inferior
	Group 2 versus 3	10.8	11	1.03	1.05	−0.20	1.04	−0.19	17.45	55.3	Inconclusive
TUG test	Group 1 versus 2	7.97	9.1	1.03	1.24	−1.13	1.135	−1.00	2.76	76	Superior
	Group 1 versus 3	7.97	10.1	1.03	1.0	−2.17	1	−2.17	1.41	93.8	Superior
	Group 2 versus 3	9.1	10.1	1.24	1.0	−1.04	1.105	−0.94	2.95	74.7	Superior
Neck tilt	Group 1 versus 2	0.11	0.55	0.12	0.19	−0.44	0.155	−2.84	1.28	98.1	Superior
	Group 1 versus 3	0.11	0.67	0.12	0.19	−0.56	0.155	−3.61	1.26	99.1	Superior
	Group 2 versus 3	0.55	0.67	0.12	0.19	−0.12	0.155	−0.77	3.68	70.7	Non-inferior
Shoulder posture	Group 1 versus 2	0.15	1.52	0.15	0.5	−1.37	0.325	−4.22	1.25	99.9	Superior
	Group 1 versus 3	0.15	1.23	0.15	0.44	−1.08	0.295	−3.66	1.25	99.5	Superior
	Group 2 versus 3	1.52	1.23	0.5	0.44	0.29	0.47	0.62	4.71	66.9	Inconclusive
Pelvic tilt	Group 1 versus 2	0.27	1.2	0.3	0.5	−0.93	0.4	−2.33	1.37	95.0	Superior
	Group 1 versus 3	0.27	1.23	0.3	0.44	−0.96	0.37	−2.59	1.32	96.6	Superior
	Group 2 versus 3	1.2	1.23	0.5	0.44	−0.03	0.47	−0.06	58.08	51.7	Inconclusive
Sensory sensitivity	Group 1 versus 2	39.1	40.6	2.33	4.62	−1.5	3.475	−0.43	7.13	61.9	Inconclusive
	Group 1 versus 3	39.1	45.1	2.33	2.6	−6	2.465	−2.43	1.34	95.7	Superior
	Group 2 versus 3	40.6	45.1	4.62	2.6	−4.5	3.61	−1.25	2.18	81.2	Superior
Sensation avoiding	Group 1 versus 2	32.9	37.5	5.64	4.06	−4.6	4.85	−0.95	2.91	74.9	Superior
	Group 1 versus 3	32.9	40.9	5.64	4.48	−8	5.06	−1.58	1.75	86.8	Superior
	Group 2 versus 3	37.5	40.9	4.06	4.48	−3.4	4.27	−0.80	3.53	71.4	Non-inferior
Sensation seeking	Group 1 versus 2	40.1	38.9	6.11	7.72	1.2	6.915	0.17	19.64	54.8	Inconclusive
	Group 1 versus 3	40.1	34.4	6.11	7.63	5.7	6.87	0.83	3.39	72.1	Non-inferior
	Group 2 versus 3	38.9	34.4	7.72	7.63	4.5	7.675	0.59	4.98	66.2	Non-inferior
Low registration	Group 1 versus 2	27.7	32.7	4.24	4.44	−5	4.34	−1.15	2.37	79.2	Superior
	Group 1 versus 3	27.7	36.7	4.24	4.16	−9	4.2	−2.14	1.14	93.5	Superior
	Group 2 versus 3	32.7	36.7	4.44	4.16	−4	4.3	−0.93	2.98	74.5	Superior
Quality of life	Group 1 versus 2	10.4	33.4	6.16	7.54	−23	6.85	−3.36	1.26	99.1	Superior
	Group 1 versus 3	10.4	61.8	6.16	11.17	−51.4	8.665	−5.93	1.25	100	Superior
	Group 2 versus 3	33.4	61.8	7.54	11.17	−28.4	9.355	−3.04	1.27	98.4	Superior

Abbreviations: NNT, necessary number for treatment; PS, probability of superiority; SD _m_ , standard deviation mean; TUG, Timed Up and Go.

Notes: x _1_ and x _2_ , means 1 and 2; sd _1_ and sd _2_ , standard deviations 1 and 2; x _1_ - x _2_ , difference between means 1 and 2.

In the *sensation avoiding* item, the clinical effect between the groups was also high (d = −0.95). This implies that 82.9% of the individuals in group 1 are above the mean of group 2, with 63.5% overlap between the groups, and a 74.9% probability of superiority for group 1. Furthermore, to achieve a more favorable outcome in group 1 compared with group 2, it is necessary to treat, on average, 2.9 individuals ( Table 1 ).

The greatest clinical effects of group 1 over group 2 were observed in items *shoulder posture* (d = −4.22), *quality of Life* (d = −3.57), and *neck tilt* (d = −2.84). This hierarchy of clinical effects is relevant for the selection of therapeutic approaches and for the planning of interventions in the continuum of care.
